# Therapeutic Targeting of Endosome and Mitochondrial Reactive Oxygen Species Protects Mice From Influenza Virus Morbidity

**DOI:** 10.3389/fphar.2022.870156

**Published:** 2022-03-23

**Authors:** Eunice E. To, Jonathan R. Erlich, Felicia Liong, Stella Liong, Raymond Luong, Osezua Oseghale, Mark A. Miles, Paris C. Papagianis, Kylie M. Quinn, Steven Bozinovski, Ross Vlahos, Robert D. Brooks, John J. O’Leary, Doug A. Brooks, Stavros Selemidis

**Affiliations:** ^1^ School of Health and Biomedical Sciences, RMIT University, Bundoora, VIC, Australia; ^2^ F.M Kirby Neurobiology Centre, Boston Children’s Hospital, Harvard Medical School, Boston, MA, United States; ^3^ Department of Pharmacology, Infection and Immunity Program, Biomedicine Discovery Institute, Monash University, Melbourne, VIC, Australia; ^4^ Cancer Research Institute and School of Pharmacy and Medical Sciences, University of South Australia, Adelaide, SA, Australia; ^5^ Sir Patrick Dun’s Laboratory, Central Pathology Laboratory, Department of Histopathology Trinity College Dublin, Dublin, Ireland; ^6^ Molecular Pathology Laboratory, Coombe Women and Infants’ University Hospital, Dublin, Ireland

**Keywords:** mitochondria, endosome, reactive oxygen, NADPH oxidase, influenza A virus, inflammation, lung inflammation, airway inflammation

## Abstract

There is an urgent need to develop effective therapeutic strategies including immunomodulators to combat influenza A virus (IAV) infection. Influenza A viruses increase ROS production, which suppress anti-viral responses and contribute to pathological inflammation and morbidity. Two major cellular sites of ROS production are endosomes *via* the NOX2-oxidase enzyme and the electron transport chain in mitochondria. Here we examined the effect of administration of Cgp91ds-TAT, an endosome-targeted NOX2 oxidase inhibitor, in combination with mitoTEMPO, a mitochondrial ROS scavenger and compared it to monotherapy treatment during an established IAV infection. Mice were infected with IAV (Hkx31 strain; 10^4^PFU/mouse) and 24 h post infection were treated with Cgp91ds-TAT (0.2 mg/kg), mitoTEMPO (100 μg) or with a combination of these inhibitors [Cgp91ds-TAT (0.2 mg/kg)/mitoTEMPO (100 μg)] intranasally every day for up to 2 days post infection (pi). Mice were euthanized on Days 3 or 6 post infection for analyses of disease severity. A combination of Cgp91ds-TAT and mitoTEMPO treatment was more effective than the ROS inhibitors alone at reducing airway and neutrophilic inflammation, bodyweight loss, lung oedema and improved the lung pathology with a reduction in alveolitis following IAV infection. Dual ROS inhibition also caused a significant elevation in Type I IFN expression at the early phase of infection (day 3 pi), however, this response was suppressed at the later phase of infection (day 6 pi). Furthermore, combined treatment with Cgp91ds-TAT and mitoTEMPO resulted in an increase in IAV-specific CD8^+^ T cells in the lungs. In conclusion, this study demonstrates that the reduction of ROS production in two major subcellular sites, i.e. endosomes and mitochondria, by intranasal delivery of a combination of Cgp91ds-TAT and mitoTEMPO, suppresses the severity of influenza infection and highlights a novel immunomodulatory approach for IAV disease management.

## Introduction

Respiratory viruses such as influenza A virus (IAV) are responsible for annual seasonal epidemics and have the potential to cause pandemic outbreaks, depending on the viral pathogenicity and level of pre-existing host immunity. IAV infection can cause debilitating respiratory illness, which poses a major concern for patients, especially high risk individuals, including the young (>age of 5), elderly (>65 years of age), pregnant, and those that are immunocompromised; notwithstanding the ongoing burden it has on global healthcare systems (Hsieh et al., 2006; La Gruta et al., 2007; Lewnard and Cobey, 2018). Currently, vaccination is the best available preventative measure for controlling and containing the spread of IAV. However, antigenic mismatches between the circulating strains and vaccines reduce the effectiveness of the vaccination approach (La Gruta et al., 2007; Houser and Subbarao, 2015; Agor and Özaltın, 2018). Prophylactic treatments include antivirals that target the viral envelope, however drug resistance is likely to limit their use in the future (Abed et al., 2005; La Gruta et al., 2007; McKimm-Breschkin, 2013; Hussain et al., 2017). Therefore, in addition to vaccination strategies, it is imperative to have therapeutic strategies that target the critical cellular machinery and signaling pathways that the virus utilizes in the host to promote inflammation and viral replication.

Reactive oxygen species (ROS), including superoxide anion and its derivatives, are highly reactive molecules that are generated by respiratory burst enzymes such as the nicotinamide adenine dinucleotide phosphate (NADPH) oxidase family of enzymes (NOX1-5, DUOX1-2), or as a consequence of cellular metabolism from the mitochondria (Wang et al., 2009; Drummond et al., 2011). ROS have important physiological roles in cellular signaling, proliferation and programmed cell death (Kannan and Jain, 2000; Redza-Dutordoir and Averill-Bates, 2016). ROS are produced by a myriad of inflammatory cells during IAV infection, including epithelial cells, monocytes, macrophages and neutrophils (Bedard and Krause, 2007; Khomich et al., 2018). Excessive ROS production to IAV infection can contribute to lung inflammation and disease pathogenesis (Imai et al., 2008; Vlahos et al., 2011). Elevations in ROS production in influenza patients were associated with higher levels of sterol oxidation products and nitro-tyrosine content, indicating changes in redox homeostasis (Nin et al., 2012; Ng et al., 2014). The specific subcellular localization of these ROS has recently emerged, with evidence showing a role for both NOX2-derived ROS from an endosomal location and mitochondrial ROS, in the pathogenesis of IAV (To et al., 2020). ROS generated from these subcellular compartments during IAV infection augment the downstream signaling pathways involved in promoting inflammation (To et al., 2017; To et al., 2019). For example, the initial internalization of virus into endosomes drives a rapid burst of ROS using a TLR7-PKC and NOX2 oxidase-dependent mechanism. This endosomal ROS response occurs within 2–5 min of virus addition to macrophages *in vitro* (To et al., 2017). This elevation in endosomal ROS also results in a marked suppression of Type I IFN that likely promotes IAV replication, and thereby exacerbating lung inflammation and oxidative stress. The molecular target of endosome ROS appears to be a single, evolutionary conserved and unique cysteine residue, C98 on TLR7, which is crucial for forming a disulphide bond with C475 and for TLR7 activation. Notably, a targeted endosomal NOX2 inhibitor, protected mice against both low and high pathogenic IAV, limiting viral replication and dramatically reducing pulmonary inflammation and alveolitis (To et al., 2017; To et al., 2019) when it was delivered to mice *prior* to IAV infection. In addition to this rapid endosomal ROS response to IAV, alterations in mitochondrial function increase mtROS (Wang et al., 2009; Makino et al., 2010; West et al., 2011; Koshiba, 2013). The temporal features of mtROS production appear to be slower in onset compared to the endosomal ROS response and are dependent on an alteration in the overall metabolic state of the immune cell(s). For instance, stimuli such as LPS stimulate an immunometabolic switch from oxidative phosphorylation to glycolysis, hours after exposure, resulting in succinate accumulation from the TCA cycle and mtROS production by Complex 1 (O’Neill and Pearce, 2016; Langston et al., 2017). Mitochondrial ROS are then required for the activation of the NLRP3 inflammasome complex (Allen et al., 2009), which is necessary for IL-1β processing and release, which promotes inflammation during IAV infection. We have recently shown that administration of the mitochondrial ROS scavenger (mitoTEMPO 36) prior to IAV infection reduced airway and lung inflammation, reduced viral mRNA and improved the overall morbidity to both low and high pathogenic influenza virus strains (To et al., 2020). In a similar fashion to endosomal NOX2 oxidase inhibition, mitoTEMPO treatment resulted in an elevation in Type I IFN and a concomitant reduction in IL-1β production. Therefore, we envisage a complex regulation of Type I IFN expression by subcellular specific ROS, involving endosome NOX2-derived ROS suppressing TLR7 directly *via* oxidation of C98, and mtROS targeting IRF7, a critical downstream regulator of TLR7 function.

It is conceivable that targeting both endosomal and mitochondrial ROS production might be an effective way of limiting the exacerbated host inflammation in response to IAV infection. Treating mice prior to IAV infection with either Cgp91ds-TAT or mitoTEMPO reduced the overall morbidity with reduced lung inflammation and improved function (To et al., 2017; To et al., 2019). However, we have up until now, no evidence that these compounds show efficacy during an established IAV infection. Therefore, in the present study we investigated the effect of the endosomal and mitochondrial ROS inhibitor 1 day after an established IAV infection. To achieve this, we delivered both Cgp91ds-TAT and mitoTEMPO simultaneously or individually 1 day post inoculation with IAV. We demonstrate that a combination of Cgp91ds-TAT and mitoTEMPO resulted in a significant reduction in airway and pulmonary inflammation, lowered the lung viral burden and elevated the number of virus-specific CD8^+^ T cells in the lung, which are important for the promotion of viral clearance. Overall, this study unravels new molecular mechanisms by which ROS promote IAV pathology and shows that subcellular specific inhibition of endosomal ROS and mtROS is a promising immunomodulatory approach against IAV infection.

## Materials and Methods

### Preparation of Virus

The IAV HKx31 (H3N2) strain was kindly provided by Prof Patrick Reading (The Peter Doherty Institute for Infection and Immunity, Department of Immunology and Microbiology, University of Melbourne). Virus stocks (6.8 × 10^8^ PFU/ml) were contained in phosphate buffered saline (PBS) and stored at −80°C until used. On the day of inoculation, aliquots of virus were thawed and diluted to the appropriate concentration.

### Cgp91 ds-tat:Ac-Asp(OChol)-PEG4-PEG3-PEG4-gp91-NH₂

Preparation of cholestanol-conjugated gp91 ds-tat (cgp91 ds-tat) was carried out by manual solid-phase peptide synthesis (SPPS) from resin-bound gp91 ds-tat, using Fmoc-PEG4-OH, Fmoc-PEG3-OH, Fmoc-PEG4-OH and Fmoc-Asp(OChol)-OH as the amino acids. After the final deprotection step, the N-terminus was capped using a mixture of acetic anhydride and N,N-diisopropylethylamine (DIPEA) in dimethylformamide (DMF) and the peptide construct was cleaved from resin using trifluoroacetic acid (TFA)/triisopropylsilane (TIPS)/1,2-ethanedithiol (EDT)/water (92.5:2.5:2.5:2.5). The crude peptide was purified as to give cgp91 ds-tat: calcd. for C173H319N56O43S (M + 5H+) m/z 780.3, obs. m/z 780.6; calcd. for C173H320N56O43S (M + 6H+) m/z 650.4, obs. m/z 650.7; calcd. for C173H321N56O43S (M + 7H+) m/z 557.6, obs. m/z 558.0 (≥95% purity).

### Chemicals

Cgp91ds-TAT was dissolved in 100% DMSO, and 2-(2,2,6,6-Tetramethylpiperidin-1-oxyl-4-ylamino)-2-oxoethyl triphenylphosphonium chloride MitoTEMPO (≥98% purity, Sigma-Aldrich) was dissolved in PBS; and prepared as a stock solution of 1 mg/ml and stored at −20°C until use. Fetal bovine serum (FBS, Sigma-Aldrich) was stored in 50 ml aliquots at −20°C. Mitosox (Thermofisher Scientific) was dissolved in DMSO (100%) at stock solutions of 1 μg/ml and stored in aliquots of 2 µL stored at −20°C.

### Animal Ethics Statement

All mouse experimentation described in this manuscript were approved by the Animal Experimentation Ethics Committee of RMIT University and conducted in compliance with the guidelines of the National Health and Medical Research Council (NHMRC) of Australia, on animal experimentation.

### *In Vivo* Infection With IAV and Intranasal Delivery of Pharmacological Agents

**FIGURE 1:**
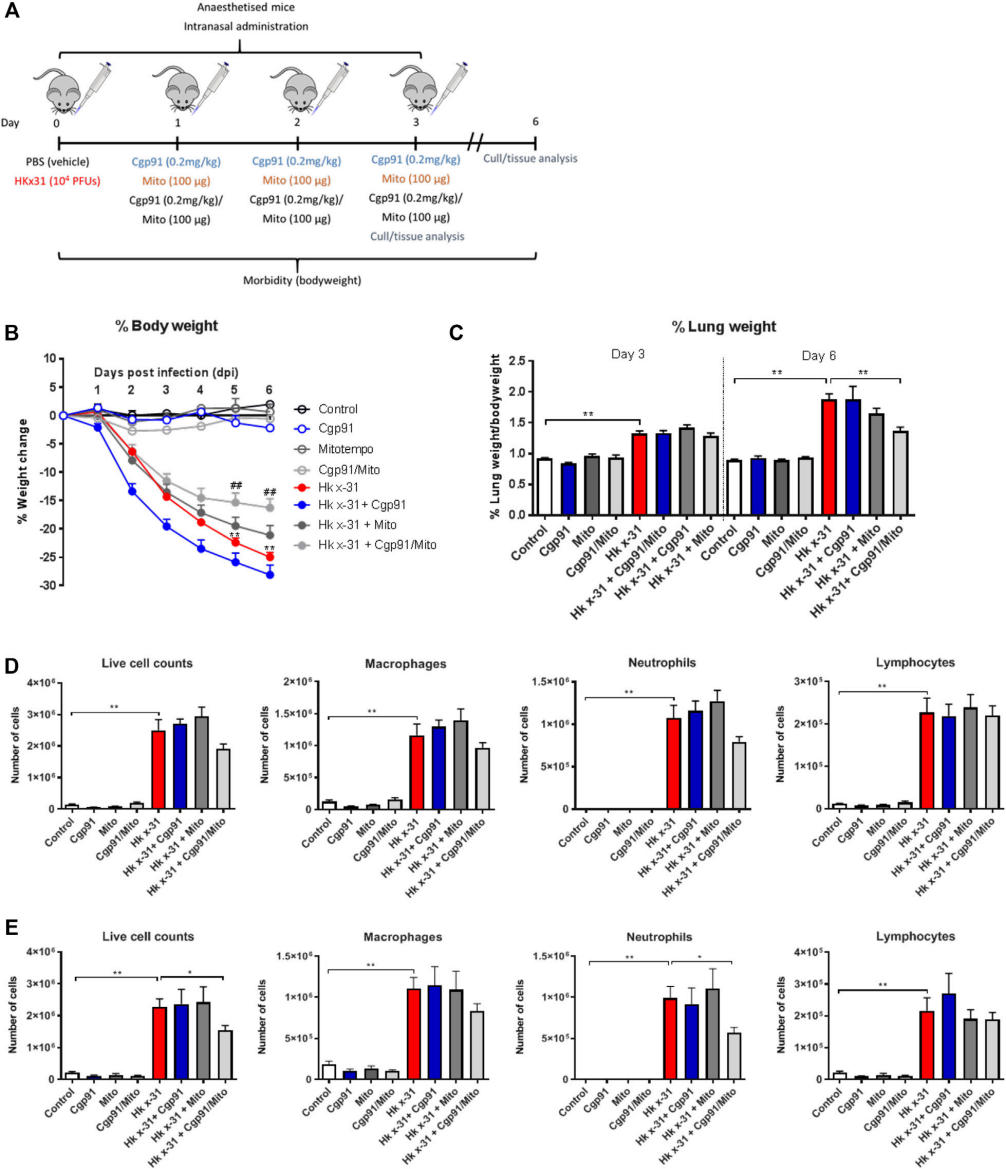
Combination therapy of Cgp91ds-TAT and mitoTEMPO reduces IAV-induced bodyweight loss, airway inflammation and pulmonary edema. **(A)** C57Bl/6J mice were treated once daily *via* intranasal administration of Cgp91ds-TAT (0.2 mg/kg) and mitoTEMPO (100 µg) in combination or individually over a 2 days period 1 day post-infection with HKx31 (10^4^ PFUs) or PBS. **(B)** Daily bodyweight measurements were taken over the experimental period. **(C)** Pulmonary edema was assessed by measuring the wet lung weight to bodyweight ratio at day 3 pi and day 6 pi. Airway inflammation was assessed *via* counting the total number of live cells isolated from the bronchoalveolar lavage fluid that were differentiated into macrophages, neutrophils and lymphocytes at **(D)** day 3 pi and **(E)** day 6 pi. 500 cells were counted from random fields by standard morphological criteria. Data are expressed as mean ± SEM (Control, *n* = 8; Cgp91, *n* = 4; mitoTEMPO *n* = 4; Cgp91/Mito, *n* = 8; Hkx31, *n* = 13; Hkx31 + Cgp91, *n* = 5; Hkx31 + Mito, *n* = 5; Hkx31 + Cgp91/Mito, *n* = 10). Statistical analysis was conducted using a two-way analysis of variance (ANOVA) followed by Holm’s Sidak post-hoc multiple comparison test in ‘B and C’ and one-way ANOVA test followed by Tukey’s post hoc test for multiple comparison test for **(D, E)**. Statistical significance was taken where *p* < 0.05 (* denotes *p* < 0.05 and **p < 0.01). ## indicates *p* < 0.01 against Hk x-31.

8–12 week-old male C57Bl/6J mice were anaesthetized by isoflurane inhalation and infected intranasally (i.n.) with 10^4^ plaque forming units (PFU) of HKx31 in a 50 µL volume of PBS. Mice were treated once daily with Cgp91ds-TAT (0.2 mg/kg) and mitoTEMPO (100 µg) (50 µL) *via* intranasal administration 1 day pi for 2 days and culled for assessment at Day 3 or 6 pi (Figure 1A). In all experiments, a randomized block experimental design was taken. Animal cages were randomly assigned a treatment group and were housed in no particular order, where the investigator would take measurements blinded to reduce the chance of bias.

### Assessment of Airways Inflammation and Differential Cell Counting

Mice were euthanised by injection (*i.p*) of a mixture of ketamine/xylazine (360 mg/kg). Bronchoalveolar lavage (BAL) differential cell counting was performed as previously described (60).

### Primary Cell Isolation

Bronchoalveolar lavage (BAL) was isolated from 8 to 12 week old male C57Bl/6J mice. Briefly, a thin shallow midline incision from the lower jaw to the top of the rib cage was made and the larynx was separated to expose the top of the trachea. The layer of smooth muscle covering the trachea was removed, a small incision was made and a sheathed 21-gauge needle was inserted into the lumen. Whilst massaging the chest, the lungs were lavaged 4 times with 300–400 µL of phosphate-buffered saline (PBS; Sigma-Aldrich, United States). The total number of viable cells in the BAL was determined by using ethidium bromide and acridine orange (Molecular Probes, United States) on a standard Neubauer hemocytometer using an Olympus BX-53 Fluorescence microscope. Cells were kept at 37°C under humidified conditions with 5% CO_2_ and 95% air.

### L-O12-Enhanced Chemiluminescence for the Detection of NOX2 Oxidase-Dependent ROS

ROS production was measured using L-012-enhanced chemiluminescence, as previously described (To et al., 2017). Inflammatory cells isolated from the BAL were seeded into a 96-well OptiView plate (5 × 10^4^ cells/well) with Dulbecco’s Modified Eagle’s Medium (DMEM; Thermofisher, United States) containing 4.5 g/L of glucose, 110 mg of sodium pyruvate and 10% Fetal Bovine Serum (FBS; Sigma-Aldrich, United States), and allowed to adhere for 3 h prior to starting the assay. Cells were then washed with warm 37°C Krebs-HEPES buffer and exposed to a Krebs-HEPES buffer containing L-012 (10^−4^ mol/L) (WAKO Chemicals) in the absence (i.e., basal ROS production) or presence (stimulated ROS production) of the protein kinase C (PKC) and NADPH oxidase activator, phorbol 12,13-dibutyrate (PDB; 10^−6^ mol/L) (Sigma-Aldrich, United States). The same treatments were performed in blank wells (i.e. with no cells), which served as controls for background luminescence. All treatment groups were performed in triplicate. Photon emission [relative light units (RLU)/s] was detected using the BMGlabtech microplate reader (CLARIOstar, Germany) and recorded from each well for 1 s over 60 cycles. Individual data points for each group were derived from the average values of the three replicates minus the respective blank controls.

### Quantification of mRNA by Quantitative PCR

Lungs were harvested from terminally anesthetized mice and tissue crushed into fine powder and total RNA extracted using an RNeasy mini kit (Qiagen, United States). Synthesis of cDNA was performed using the High-Capacity cDNA RT kit (P/N4322171, Life Technologies, Foster City, CA, United States) using 1.0–3.0 µg total RNA. Quantitative polymerase chain reaction was carried out using the TaqMan Fast advanced Master Mix (Life Technologies, Foster City, CA, United States) or SYBR Green PCR Master Mix (Life Technologies, Foster City, CA, United States) and analyzed on the QuantStudio seven Flex Real-Time PCR system (Life Technologies). The PCR primer for IFN-β, was included in the Assay-on-Demand Gene Expression Assay Mix (Life Technologies, Foster City, CA, United States). Additionally, a custom designed forward and reverse primer of the segment three polymerase (PA) of IAV was used to measure viral load. The PCR program run settings were: 50°C for 2 min, followed by 95°C for 1 h, then 95°C for 15 s + 60°C for 60 s + plate read (40 cycles). For Fast Advanced Master Mix, the program settings were: 50°C for 2 min, 95°C for 2 min, 95°C for 1 s, 60°C for 20 s + plate read (40 cycles). Quantitative values were obtained from the threshold cycle (Ct) number. Target gene expression was normalized against glyceraldehyde 3-phosphate dehydrogenase (GAPDH) mRNA expression for each sample and data was expressed relative to the naïve control group.

### Histology

The left lung was dissected from mice and immersed in neutral buffered formalin (10%) for 24–48 h. After fixation, the lung tissue was processed, embedded in paraffin wax, and longitudinal 4 μm sections cut and stained with hematoxylin and eosin (H&E). Slides were scanned by light microscopy and uploaded to Aperio microscope scanner (Leica Biosystems, Nussloch, Germany). Histology was performed by the Department of Histology (Monash University, Clayton, Australia) and analysed blindly by two independent assessors using an Aperio Imagescope. Each sample was scored from 0–5 for each individual mouse (higher numbers indicate increased severity). Five random fields from each lung section was analysed for alveolitis, which is inflammation within the alveolar space. This was determined by the regularity and branching of the alveoli and the density of cells within the alveolar spaces. Peribronchiolar inflammation was characterised by the infiltration of inflammatory cells into the alveolar wall around the bronchioles. The degree of inflammatory cellular infiltrate was taken by observing the density of cells throughout the entire lung section.

### Flow Cytometry and Tetramer Staining

Whole lung was finely minced with scissors and enzymatically digested using liberase (Sigma-Alrich, United States). Single cell suspensions were prepared from homogenised tissue straining through a 40 μM mesh filter. The red blood cells were lysed with lysis buffer (ACK lysis buffer) and the WBC stained with respective fluorescent-labelled anti-mouse antibodies for flow cytometric analysis: leukocytes (CD45; 30-F11), T cells CD3 (145-2C11), CD8 (53–6.7), activation marker (CD69; H1.2F3) and neutrophils (Ly6G; 1A8) (Invitrogen, United States). Tetramer staining of virus-specific CD8^+^ T cells was performed using the peptides D^b^NP_366_ (ASNENMETM) that was synthesised at Biomolecular Resource Facility, Australian National University, Australia. Within each antibody cocktail mixture, cells were incubated with CD16/32 (2.4G2) to block Fc-mediated adherence of the antibodies. Cell viability was determined by LIVE/DEAD Fixable Violet Dead Cell Stain Kit (Invitrogen). Total mitochondrial ROS was assessed using the fluorescent dye-based MitoSOX™ Red with the absorption/emission: 510/580 nm. A minimum of 100,000 events were acquired for each sample on the FACSAria II (BD Biosciences, United States) and data analysis carried out using FlowJo (United States) software. Manual clustering of multidimensional flow cytometry was guided by isotype controls and/or untreated samples.

### Statistical Analysis and Image Analysis

All results are presented as mean ± SEM. Statistical analysis of Figure 1B was performed using a two-way analysis of variance (ANOVA) followed by Holm’s Sidak post-hoc test. Analysis of all the other figures were conducted using a one-way ANOVA followed by Tukey’s post hoc test for multiple comparison. All statistical tests were performed using GraphPad Prism (GraphPad Software Version 7.0, San Diego CA, United States) where *p* < 0.05 was taken to indicate significance.

## Results

### The Combination of Cgp91ds-TAT and mitoTEMPO is More Effective Than Monotherapy Treatment in Reducing IAV-Induced Bodyweight Loss, Mortality and Airway Inflammation

One of the key indicators of disease severity during IAV infection is a reduction in bodyweight. In the absence of any treatment, mice infected with 10^4^ PFUs of IAV lost ∼15% of their bodyweight at 3 days pi and ∼24% at 6 days pi. Treatment with the combination of the endosomal and mitochondrial ROS inhibitors significantly (*p* < 0.01) reduced bodyweight loss at days 5–6 pi (Figure 1B), whereas the individual treatments had no significant effect on the bodyweight loss caused by IAV infection. The vehicle-treated naïve animals did not display alterations in bodyweight over the course of the experimental procedure (Figure 1B). The lung wet weight to bodyweight ratio was used to assess the impairment in alveolar fluid clearance that contributes to oedema. Influenza virus infection caused an increase in oedematous lungs at day 3 pi and was further increased at day 6 pi compared to the PBS uninfected mice (Figure 1C). Treatment with the combination of ROS inhibitors did not alter this response at day 3 pi, but reduced the lung to bodyweight ratio at day 6 pi (Figure 1C). Notably, the individual use of the ROS inhibitors did not alter the weight of the lung tissue at both time points. The drug treatments in naïve mice had no effect on baseline lung weight to bodyweight ratios (Figure 1C). A scrambled version of Cgp91ds-TAT was previously shown not to influence the IAV-dependent inflammatory response in mice (To et al., 2017).

To evaluate airway inflammation within the respiratory tract, the total number of live cells in the BALF were counted. Intranasal inoculation with IAV caused an increase in airway immune cell infiltration at Days 3 and 6 pi (Figures 1D,E, respectively). Treatment with the combination of Cgp91ds-TAT and mitoTEMPO caused a trend for reduction in airway inflammation and neutrophil infiltration, but had no effect on lymphocyte or macrophage numbers at day 3 pi (Figure 1D). At day 6 pi, the combination of Cgp91ds-TAT and mitoTEMPO caused a significant (*p* < 0.05) reduction in airway inflammation and neutrophilic inflammation, although the treatment did not alter the macrophage or lymphocyte count (Figure 1E). Comparatively, monotherapy treatment did not significantly alter airway inflammation or differential cell analysis at both time points (Figures 1D,E). In uninfected cohorts, a combination of Cgp91ds-TAT and mitoTEMPO or monotherapy had no effect on total airway cellularity (Figures 1D,E).

### Therapeutic Administration of Cgp91ds-TAT and mitoTEMPO Alleviates Pulmonary Inflammation

**FIGURE 2:**
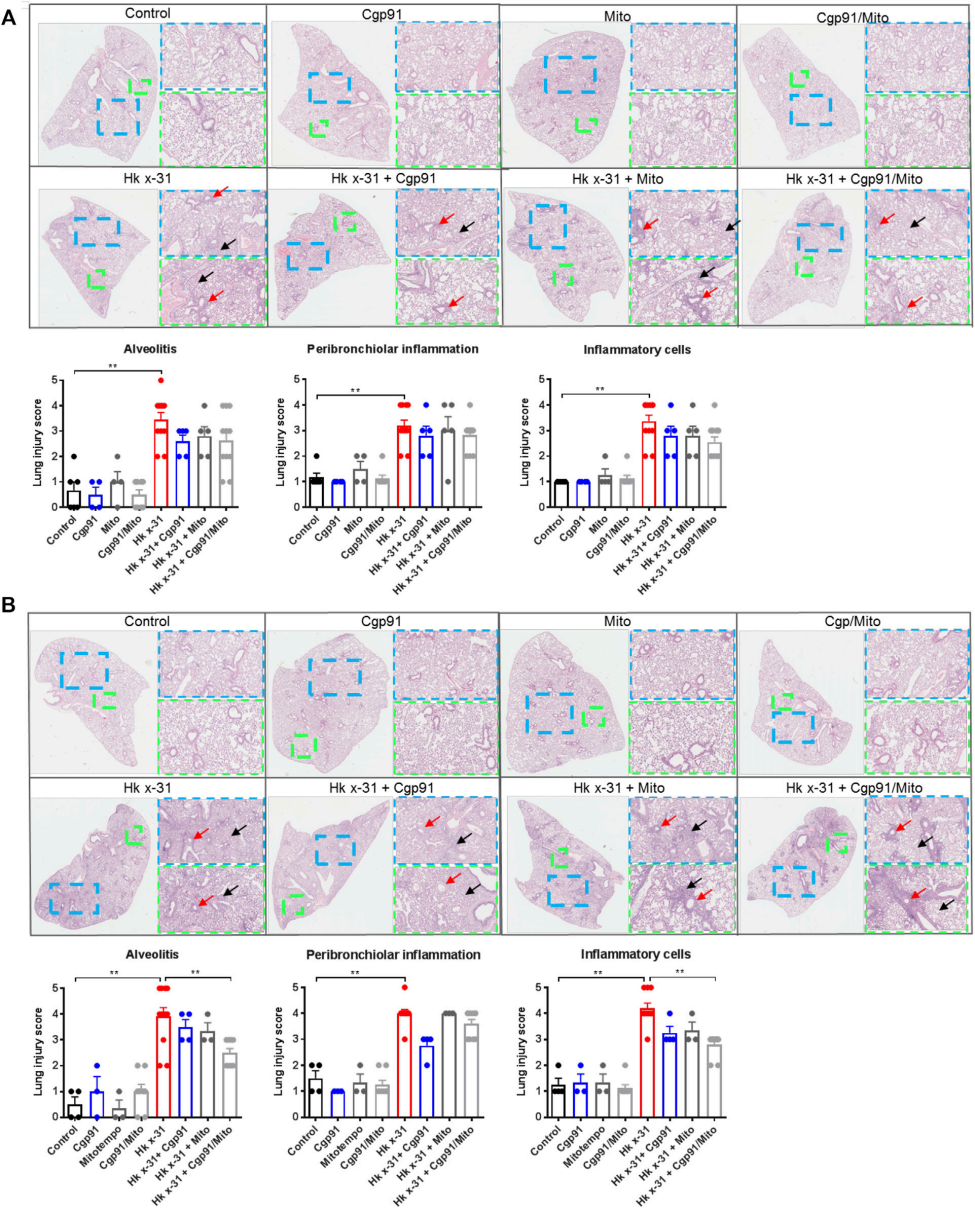
Dual inhibition of endosomal and mitochondrial ROS reduces lung pathology in IAV infected mice. Histopathological analysis of lungs from C57Bl/6J mice that were infected with Hkx31 (10^4^ PFUs) or PBS *via* intranasal administration. Animals were treated once daily 1-day following infection *via* intranasal administration with Cgp91ds-TAT (0.2 mg/kg) and mitoTEMPO (100 µg) in combination or individually over a 2 days period at **(A)** day 3 pi and **(B)** day 6 pi. Representative images displaying lung inflammation from paraffin embedded lungs were sectioned (10 μm) longitudinally and stained with H&E. Each sample was scored blindly from 0–5 for each individual mouse (higher numbers indicate increased severity) from two independent assessors. Sections were scored for alveolitis (red arrows), inflammatory cell infiltrate and peribronchiolar inflammation (black arrows). Representative images are presented at three different magnifications [×1 (clear), ×3 (blue), ×6 (green)]. Data are expressed as mean ± SEM. Statistical analysis was conducted using non-parametric Mann-Whitney test. Statistical significance was taken where *p* < 0.05 (* denotes *p* < 0.05 and **p < 0.01).

Pulmonary inflammation due to an infiltration of immune cells is a key feature that contributes to lung injury during IAV infection. To assess the histological changes, lung sections were scored blindly for measures of airway and lung inflammation. Infection with IAV caused extensive peribronchiolar inflammation, increased cellular infiltration in the alveolar space and an increased number of inflammatory cells compared to the naïve uninfected mice at day 3 pi (Figure 2A). On day 6 post-inoculation, these measures of lung inflammation and lesions were more pronounced than at day 3, as expected (Figure 2B). Treatment with either Cgp91ds-TAT or mitoTEMPO alone or in combination had no effect on the histopathological changes induced by IAV infection at the Day 3 time point (Figure 2A). At day 6 pi, Cgp91ds-TAT treatment caused a trend to lower all the parameters measured but statistical significance was not reached. Similarly, animals treated with mitoTEMPO displayed modest but statistically insignificant reductions in alveolitis and overall inflammatory cellular infiltration, but did not affect peribronchiolar inflammation (Figure 2B). In contrast, a combination of Cgp91ds-TAT and mitoTEMPO significantly reduced alveolitis and cellular infiltrates, but had no impact on peri-bronchiolitis (Figure 2B). Notably, there was no observable inflammation due to administration of Cgp91ds-TAT, mitoTEMPO or the combination of Cgp91ds-TAT, mitoTEMPO in uninfected animals (Figures 2A,B).

### Dual Cgp91ds-TAT and mitoTEMPO Administration Abrogates Mitochondrial ROS and Alters NOX2 Oxidase-Derived ROS

**FIGURE 3:**
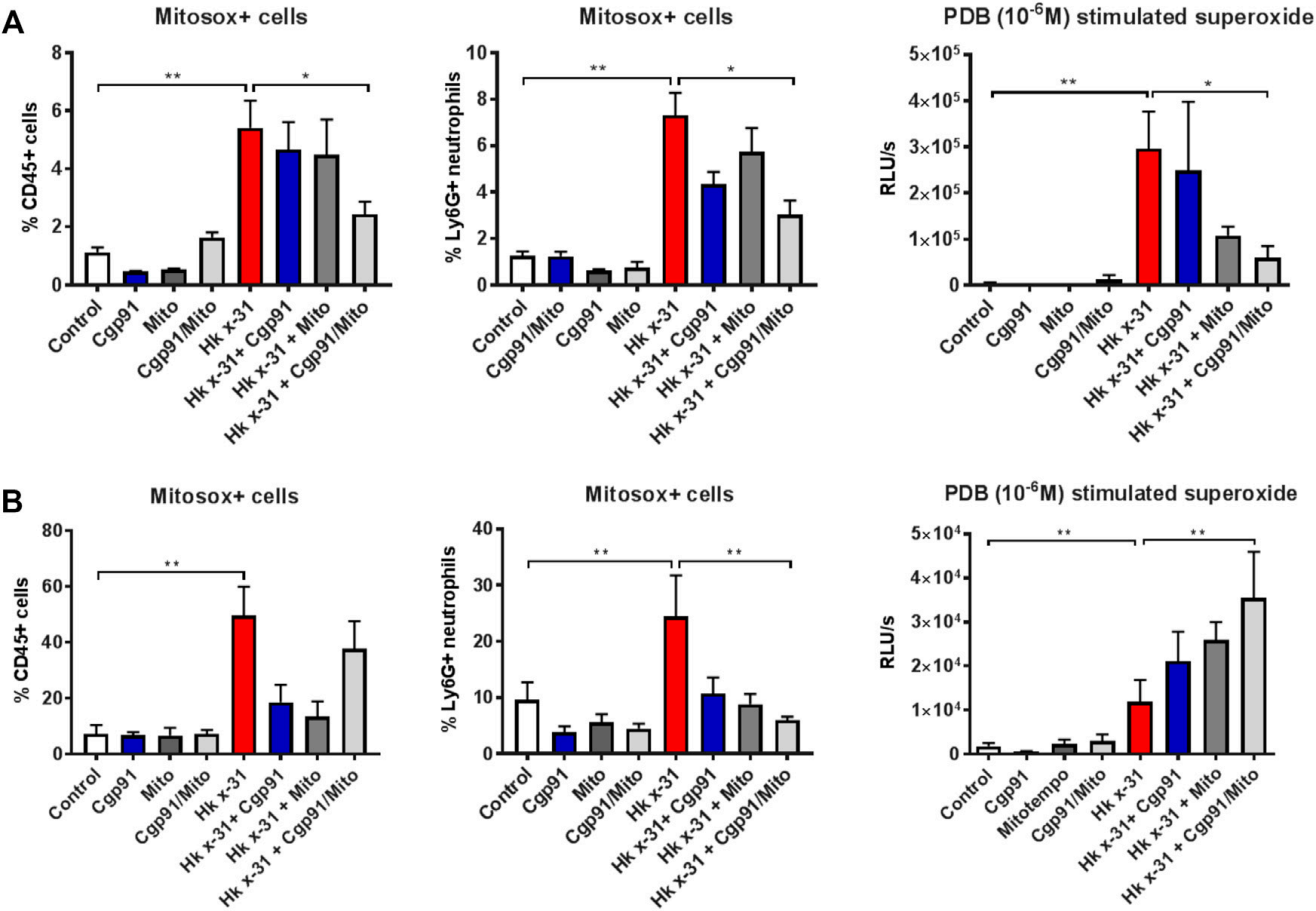
Effects of ROS inhibition on mitochondrial and NOX2-derived ROS generation. C57Bl/6J mice were treated once daily *via* intranasal administration of Cgp91ds-TAT (0.2 mg/kg) and mitoTEMPO (100 µg) in combination or individually over a 2 days period 1 day pi with Hkx31 (10^4^ PFUs) or PBS for assessment at **(A)** day 3 pi and **(B)** day 6 pi. Cells isolated from lung tissue were collected for mitochondrial ROS measurements by staining cells with a fluorescent probe (Mitosox) and assesed using flow cytometric analysis. The populations are measured as a % of mitosox positive cells gated from the CD45^+^ population. The percentage of Mitosox + cells were further characterised in Ly6G + neutrophils by gating from the CD45^+^ population. PDB (10^−6^ M) stimulated ROS production that was quantified by L-O12 enhanced chemiluminescence from BALF inflammatory cells. Data are expressed as mean ± SEM (Control, *n* = 6; Cgp91, *n* = 4; mitoTEMPO *n* = 4; Cgp91/Mito, *n* = 6; Hkx31, *n* = 8 Hkx31 + Cgp91, *n* = 5; Hkx31 + Mito, *n* = 5; Hkx31 + Cgp91/Mito, *n* = 8). Statistical analysis were conducted using one-way ANOVA test followed by Tukey’s post hoc test for multiple comparison test. Statistical significance was taken where *p* < 0.05 (* denotes *p* < 0.05 and **p < 0.01).

Flow cytometric analysis was used to determine the effectiveness of the ROS inhibitors in suppressing mtROS and NOX2-derived ROS in lung tissue. IAV infection increased the percentage of mitoSOX^+^ CD45^+^ cells in the lung tissue at day 3 pi compared to the naïve controls (Figure 3A). This response was significantly reduced with the combination of Cgp91ds-TAT and mitoTEMPO but unaffected by monotherapy treatment at day 3 pi, indicating intranasal drug administrations can effectively inhibit mtROS at the direct site of infection (i.e., in the lungs). Similarly, the percentage of mitoSOX + neutrophils (Ly6G^+^) in the lung at this time point was attenuated with the combination of drugs, but not by individual treatments (Figure 3A).

At Day 6 pi, the mitoSOX^+^ CD45^+^ population was significantly elevated however, this was unaffected by the combination of Cgp91ds-TAT and mitoTEMPO. Interestingly, the individual ROS inhibitors were able to reduce this response (Figure 3B). Moreover, treatment with both monotherapy and combined therapy effectively prevented the increase in mitoSOX^+^ neutrophils back to similar levels of the uninfected control groups (Figure 3B).

L-012-enhanced chemiluminescence was used to quantify NOX2 oxidase-dependent ROS generation in BAL isolated from mice. BAL cells taken from IAV infected mice exhibited a significant (*p* < 0.01) increase in ROS production compared to naïve mice that was blunted with combined drug treatment at Day 3 pi. At this time point, Cgp91ds-TAT did not alter the response, but mitoTEMPO caused a reduction in IAV-induced ROS production (Figure 3A). At day 6 pi, combined therapy significantly (*p* < 0.01) increased ROS generation in comparison to the virus control group, but this response was unchanged with monotherapy treatment (Figure 3B). NOX2-derived ROS levels were unaltered in BAL inflammatory cells taken from naïve mice treated with the ROS inhibitors (Figures 3A,B).

### Temporal Changes in IFN-β Gene Expression and Viral Load With ROS Inhibition

**FIGURE 4:**
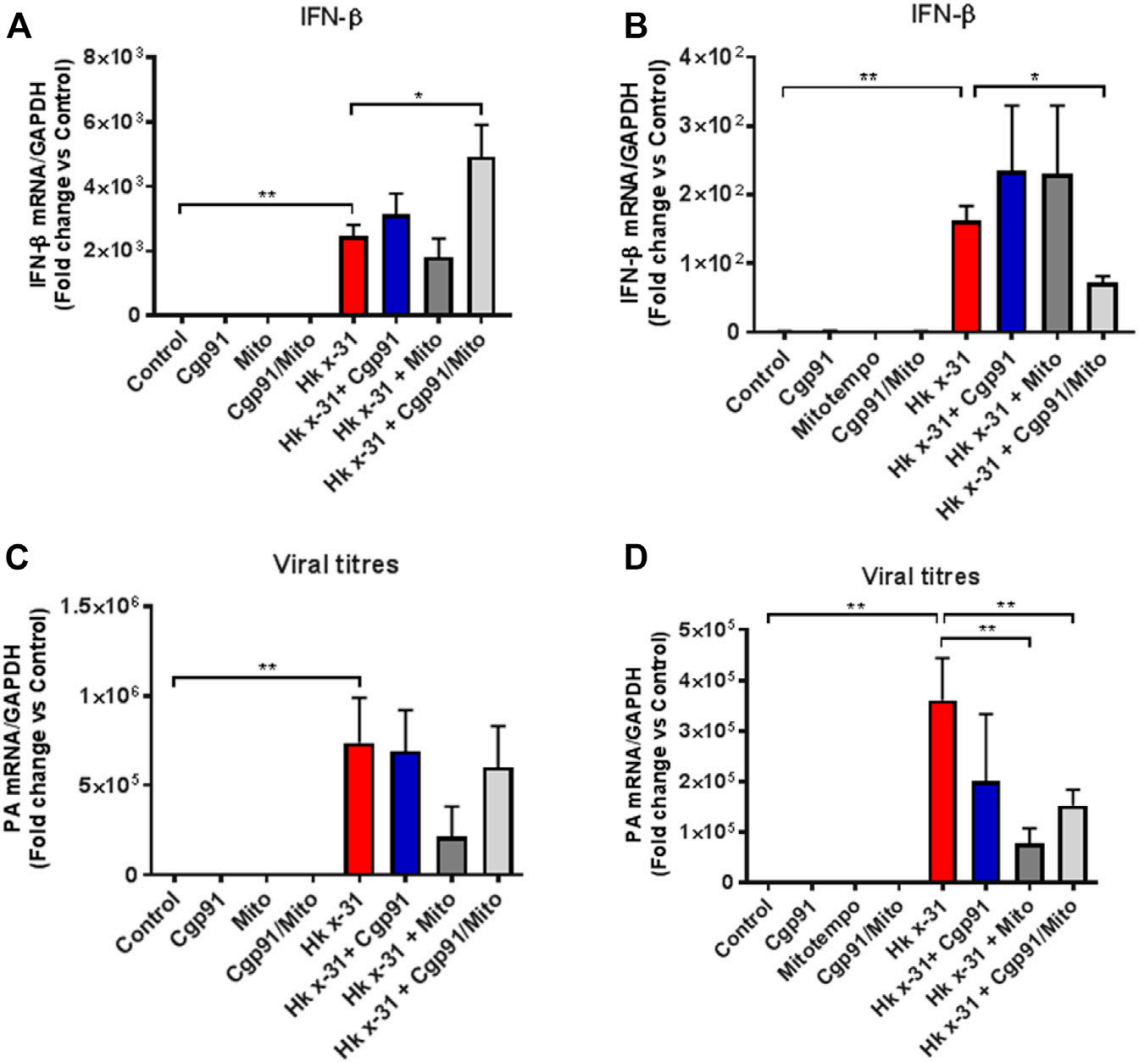
Lung IFN-β and viral mRNA expression at an early and late stage of influenza A virus infection. Mice were infected with Hx x-31 at (10^4^ PFUs) or PBS *via* intranasal administration. Animals were treated once daily 1 day following infection *via* intranasal administration with a combination of Cgp91ds-TAT (0.2 mg/kg) and mitoTEMPO (100 µg) or ROS inhibitors alone over a 2 days period for IFN-β analysis at **(A)** day 3 pi and **(B)** day 6 pi. and mRNA analysis of the gene encoding polymerase of influenza virus strain by QPCR in **(C)** at day 3 pi and **(D)** day 6 pi. IFN-β and viral PA were quantified in lung tissue. Responses are relative to GAPDH and expressed as a fold-change above naïve controls. Data are expressed as mean ± SEM (Control, *n* = 6; Cgp91, *n* = 4; mitoTEMPO *n* = 4; Cgp91/Mito, *n* = 8; Hkx31, *n* = 14 Hkx31 + Cgp91, *n* = 5; Hkx31 + Mito, *n* = 5; Hkx31 + Cgp91/Mito, *n* = 12). Statistical analysis was conducted using one-way ANOVA test followed by Tukey’s post hoc test for multiple comparison. Statistical significance was taken where *p* < 0.05 (* denotes *p* < 0.05 and **p < 0.01).

Severe IAV infections are often associated with anti-viral and pro-inflammatory cytokine generation. To determine the effect of endosomal and mitochondrial ROS inhibition on IAV-dependent pro-inflammatory cytokine expression, we measured the expression of antiviral Type I IFN in the lungs *via* qPCR. The expression of IFN-β was significantly (*p* < 0.05) elevated at days 3 and 6 pi in mice challenged with IAV compared to the naïve controls (Figures 4A,B). At day 3 pi, mice treated with Cgp91ds-TAT and mitoTEMPO in combination, but not monotherapy displayed a further enhancement in IFN-β (Figure 4A). In contrast, at day 6 pi., mice treated with Cgp91ds-TAT and mitoTEMPO in combination displayed a reduced level of IFN-β (Figure 4B).

We next examined the viral load in the lungs by analyzing the mRNA of the gene encoding segment three polymerase of IAV. At both day 3 and 6 pi, the amount of IAV mRNA was substantially higher compared to the naïve control mice (Figures 4C,D). Neither the combination treatment nor individual treatment altered the viral mRNA expression compared to the virus-infected vehicle cohort at day 3pi (Figure 4C). In contrast, at the day 6 pi time point, the viral mRNA level was significantly (*p* < 0.01) lower in mice treated with a combination of Cgp91ds-TAT and mitoTEMPO or with mitoTEMPO alone, but not with Cgp91ds-TAT alone (Figure 4D).

### Combined Therapy Elevates the Number of Virus-Specific CD8^+^ T Cells

**FIGURE 5:**
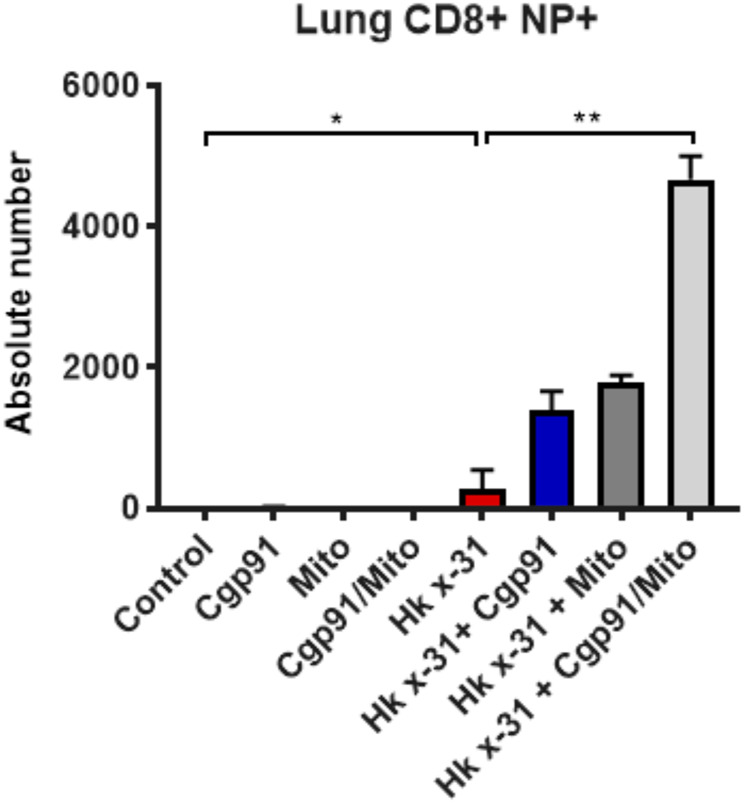
Combination therapy elevates virus-specific T cells in the lung. C57Bl/6J mice were treated once daily *via* intranasal administration of Cgp91ds-TAT (0.2 mg/kg) and mitoTEMPO (100 µg) in combination or individually over a 2 days period 1 day pi with Hkx31 (10^4^ PFUs) or PBS for assessment at day 6 pi. Cells isolated from the lung tissue were collected for immune cell differentiation that was assesed using flow cytometric analysis. Virus-specific CD8^+^ T cells were gated as CD8+NP366+. Data are expressed as mean ± SEM (Control, *n* = 4; Cgp91, *n* = 4; mitoTEMPO *n* = 4; Cgp91/Mito, *n* = 4; Hkx31, *n* = 5 Hkx31 + Cgp91, *n* = 6; Hkx31 + Mito, *n* = 6; Hkx31 + Cgp91/Mito, *n* = 6). Statistical analysis were conducted using one-way ANOVA test followed by Tukey’s post hoc test for multiple comparison test. Statistical significance was taken where *p* < 0.05 (* denotes *p* < 0.05 and **p < 0.01).

Flow cytometric analysis was used to assess the predominant epitope (NP_366–374_) recognized by influenza virus-specific CD8^+^ T cells. Virus infection resulted in an elevation in a population of CD8+NP + T cells in the lung that was further increased by the combination of Cgp91ds-TAT and mitoTEMPO (Figure 5). Monotherapy treatment also enhanced the number of CD8+NP + T cells compared to the virus-infected vehicle-treated cohort (Figure 5).

## Discussion

IAV infections trigger respiratory tract complications, which feature heightened oxidative burden due to excessive ROS generation, resulting in exacerbated lung inflammation and injury (Vlahos et al., 2011). NOX2 oxidase-derived ROS contributes to lung pathology during IAV infection and endosomes are a major site for this ROS generation (Snelgrove et al., 2006; Vlahos et al., 2011; To et al., 2017; To et al., 2019). ROS produced by the mitochondria is also a major contributor to IAV pathogenesis (Dikalova et al., 2010; Kim et al., 2015). In this present study, we demonstrated that acute inhibition of ROS in both subcellular compartments (mitochondria and endosomes) was effective in reducing airway and pulmonary inflammation and in limiting viral load following IAV infection. Collectively, these data indicate that the targeting of ROS in these distinct compartments is a novel immunomodulatory approach for the treatment of IAV infections.

We demonstrated either no effect or a rather modest reduction in the disease course in mice treated with a combination of Cgp91ds-TAT and mitoTEMPO following IAV infection at Day 3 post infection. However, the effects of Cgp91ds-TAT and mitoTEMPO treatment were significantly more pronounced at Day 6 post infection. The phenotype of the treated mice was characterised by a reduction in BALF inflammation and neutrophilia, suppression in bodyweight loss, inhibition of lung oedema and a reduction in viral load signifying an overall improved disease course following treatment. The delayed improvement in disease course by ROS inhibition exemplifies that ROS are important regulators of the immune response to IAV infection, and that the dampening of their actions can be an effective means in re-shaping the inflammatory response to IAV. Often the inflammatory response to IAV is uncontrolled and exacerbated resulting in a significant lung immunopathology that governs the overall degree of sickness with impacts on both the innate and adaptive immune control of the viral burden. Our study demonstrates that both endosomal and mitochondrial ROS inhibition resulted in a reduced lung inflammatory response and a more effective anti-viral CD8^+^ T cell response. It is important to indicate that our study is only a proof of concept that the dual inhibition of endosomal ROS and mitochondrial ROS is effective at reducing IAV pathogenesis. The study is not a dose-finding study for either mitoTEMPO or Cgp91ds-TAT. Each drug on its own demonstrated very modest effects at re-shaping the immune response to IAV. This could signify a great deal of redundancy in the cellular effects of mtROS and endosomal ROS and that effective control can only occur when both pools of ROS are suppressed. Alternatively, it might signify that the dosing regimen of drugs chosen i.e., 3 days treatment protocol might be sub-optimal. Another important observation is that the endosomal targeted Cgp91ds-TAT did not suppress the PDB-dependent NOX2 activity indicating that Cgp91ds-TAT allows for intact extracellular superoxide production by NOX2 but rather provides a site specific targeting of superoxide in endosomes. This is critical, as elements of NOX2 activity remaining functional, is important for clearance of bacteria. The co-operative effects of the two drugs suggest a potential interplay between mitochondrial and endosomal ROS. Indeed, in support of such interplay, NOX2 has been shown to induce mitochondrial superoxide production in angiotensin-II mediated endothelial oxidative stress during hypertension (Dikalov et al., 2014). Certainly, crosstalk between these important sources of ROS including with other sources such as NOX4 and Duox enzymes warrants further investigation.

It is interesting that when using a *prophylactic* drug approach with either Cgp91ds-TAT or mitoTEMPO alone, the pathology to IAV infection was significantly dampened (To et al., 2017; To et al., 2019). Thus, suppression of either endosomal ROS or mitochondrial prior to IAV infection effects a different immune response that improves the disease course in mice. Intriguingly in both previous studies, we showed that either Cgp91ds-TAT or mitoTEMPO treatment alone in mice prior to IAV infection resulted in a significantly greater anti-viral Type I IFN, i.e., IFN-β response to IAV. In contrast in the present study, the administration of either Cgp91ds-TAT or mitoTEMPO alone post-IAV infection had no effect on Type I IFN, however, only with the combination treatment did we observe a significant elevation in Type I IFN. Again, this indicates the complex nature and timing of IFN signaling in response to IAV and how modifiable this signaling is to ROS suppression.

The Type I IFN system is upregulated during IAV infection and regarded as one of the most powerful innate defenses against viral pathogens (Khaitov et al., 2009; Crotta et al., 2013). Here we demonstrate that ROS inhibition causes an elevation in IFN-β expression at the early stages of infection. Pattern recognition receptors, such as toll-like receptors have been implicated in the induction of IFNs during IAV infection. In particular, TLR7 detects single stranded RNA components of IAV in endosomes and induces IFN secretion (Lund et al., 2004; Diebold et al., 2015; To et al., 2017). We have previously unveiled a feedback loop where endosomal NOX2-derived ROS suppressed TLR7 activity (To et al., 2017). Similarly, an elevation in mtROS reduced type I IFN production markedly *via* inhibiting the phosphorylation of interferon regulatory factor 7 (IRF7), which is a key transcription factor for TLR7 signaling (Agod et al., 2017). Based on these findings, it appears that there is a synergistic effect in targeting both endosomal and mtROS to boost a protective IFN response at the early phase of infection. Despite an increase in type I IFN responses with the dual ROS inhibition strategy, this did not correlate with a reduction in viral load at day 3 pi but it was significantly suppressed at Day 6 pi. This may be due to a delayed manifestation of the anti-viral effects of the increased IFN response. The reduction in viral titres was associated with a reduction in IAV-induced bodyweight loss, which is likely due to the overall reduced lung inflammation. From these observations, it can be postulated that both mitochondrial and endosomal ROS negatively regulate anti-viral cytokine generation. Although the exact mechanisms underlying this phenomenon are not yet characterized, it is tempting to propose that ROS causes oxidation of respiratory chain proteins that affect metabolism and pattern recognition receptors, such as endosomal TLR7, which have been implicated in ROS biology (To et al., 2017).

Both mtROS and NOX2 oxidase-derived ROS were significantly blunted in lung inflammatory cells with daily drug administration of a combination of Cgp91ds-TAT or mitoTEMPO, validating the effectiveness of this approach in dampening ROS production. This is likely due to a reduction in airway neutrophils, as they are a major source of ROS (Dupré-Crochet et al., 2013). Mechanistically, ROS inhibition reduces the recruitment of neutrophils, most likely by lowering the expression of the chemokine receptor CXCL2. Indeed, CXCL2 antagonism protected mice against respiratory IAV and pneumococcal infections, suggesting neutrophilia contributes to a worsening of the pathological condition during influenza infection (Perrone et al., 2008; Tavares et al., 2017). Moreover, another study revealed that the lack of the antioxidant defense enzyme nuclear factor erythroid 2–related factor 2 (NRF-2) caused persistent inflammatory cell infiltration into the lung with elevated levels of CXCL2 expression during hyperoxia-induced acute lung injury (Reddy et al., 2009). However, there is evidence to demonstrate that neutrophils are necessary for host recovery (Fujisawa, 2008). In fact, neutrophil-depleted mice exhibited exacerbated levels of pulmonary inflammation and respiratory dysfunction during IAV infection (Tate et al., 2008; Tate et al., 2009), indicating that an excessive and uncontrolled neutrophilic inflammation precedes mortality in mice. These findings indicate a balance of the pro-oxidative capacity and antioxidant defense system needs to be tightly regulated to avoid complete ablation of neutrophils, as basal levels are necessary for pathogen killing, but too many neutrophils contribute to pathological inflammation owing to their high capacity for ROS production.

Airway and lung parenchymal inflammation are pathological characteristics of IAV and in the present study, IAV infection caused significant and extensive alveolitis, peribronchiolar inflammation and infiltrating inflammatory cells, together with airway epithelial denudation and peri-vascular inflammation. The combination of Cgp91ds-TAT and mitoTEMPO treatment was more effective than monotherapy treatment in attenuating alveolitis and to a lesser degree peribronchiolar inflammation, indicative that ROS produced from these subcellular compartments synergistically exacerbate the resultant pulmonary damage during influenza virus infection. Concurrently, this correlated with an improvement in lung oedema. A key characteristic that perpetuates the formation of lung oedematous tissue during influenza virus challenge is the impairment of the alveolar fluid clearance (Wolk et al., 2008). Interestingly, this deterioration in alveolar fluid clearance was reversed with a protein kinase C inhibitor, which phosphorylates the subunits on NADPH NOX enzymes, thus preventing the culmination of ROS production. This suggests that ROS are a major contributor to the deleterious parenchymal inflammation during IAV infection and that suppression of ROS might be an effective means of minimizing pathological parenchymal inflammation, whilst retaining some level of peri-bronchial inflammation for effective viral clearance. Importantly, targeting these two ROS compartments did not result in a global immunosuppressive phenotype but rather specific elements of the inflammatory response were modulated(Dikalov et al., 2014).

## Conclusion

This is the first study to investigate intranasal delivery of a combination of an endosomal and mitochondrial ROS inhibitor as a therapeutic strategy against IAV infection. Intranasal delivery of ROS inhibitors to effectively reduce influenza immunopathology provides a potential therapeutic strategy to reduce the severity of IAV infection. The advent of this novel technology in inhibiting compartmentalized ROS not only advances the fundamental knowledge of how ROS-dependent processes influence IAV pathogenesis, but also highlights exciting and novel interventional strategies for the treatment of IAV infections, which is independent of the infecting viral strain, and appropriate for when annual vaccinations and antivirals fail to offer protection.

## Data Availability

The original contributions presented in the study are included in the article/Supplementary Material, further inquiries can be directed to the corresponding author.
